# Bis[1,3-bis­(2,4,6-trimethyl­phen­yl)-2,3-dihydro-1*H*-imidazol-2-yl­idene]dinitros­yl(tetra­hydro­borato-κ^2^ *H*,*H*′)tungsten(0)

**DOI:** 10.1107/S1600536810052426

**Published:** 2010-12-18

**Authors:** Javier Fraga-Hernández, Olivier Blacque, Heinz Berke

**Affiliations:** a Institute of Inorganic Chemistry, University of Zürich, Winterthurerstrasse 190, 8057 Zürich, Switzerland

## Abstract

In the title paramagnetic 19-electron neutral complex, [W(BH_4_)(C_21_H_24_N_2_)_2_(NO)_2_], the W(0) atom is coordinated by two 1,3-bis­(2,4,6-trimethyl­phen­yl)imidazol-2-yl­idene (IMes) carbene ligands, two NO groups and two H atoms of an η^2^-tetra­hydro­borate ligand. Depending on the number of coordination sites (*n*) assigned to the BH_4_^−^ ligand, the coordination geometry of the W atom may either be described as approximately trigonal–bipyramidal (*n* = 1) or as very distorted octa­hedral with the bridging H atoms filling two coordination positions (*n* = 2). In the latter case, the coplanar NO groups and bridging H atoms (r.m.s. deviation = 0.032 Å) form one octa­hedral plane, with mutually *trans*-oriented carbene ligands. In the crystal, mol­ecules are connected *via* C—H⋯O inter­actions.

## Related literature

For the synthesis, characterization and reactivity of dinitrosyl tungsten complexes in various oxidation states, see: Fraga-Hernández (2007 ▶). For a related complex with the W(NO)(η^2^-BH_4_) core, see: van der Zeijden *et al.* (1991 ▶). For tungsten complexes with *N*-heterocyclic (NHC) carbenes, see: Nonnenmacher *et al.* (2005 ▶); Hahn *et al.* (2005 ▶); Wu *et al.* (2007 ▶); Fraga-Hernández *et al.* (2011 ▶). For an overview of the first organometallic nitro­syls known, see: Enemark & Feltham (1974 ▶); Richter-Addo & Legzdins (1988 ▶); Berke & Burger (1994 ▶).
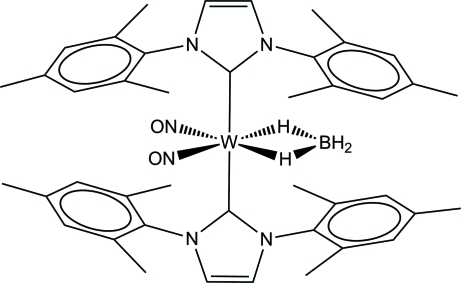


## Experimental

### 

#### Crystal data

[W(BH_4_)(C_21_H_24_N_2_)_2_(NO)_2_]
*M* *_r_* = 867.56
Monoclinic, 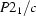

*a* = 24.7322 (13) Å
*b* = 11.2183 (5) Å
*c* = 15.0522 (8) Å
β = 97.643 (6)°
*V* = 4139.2 (4) Å^3^
*Z* = 4
Mo *K*α radiation
μ = 2.83 mm^−1^
*T* = 183 K
0.30 × 0.20 × 0.18 mm

#### Data collection

Stoe IPDS diffractometer
Absorption correction: numerical (Coppens *et al.*, 1965 ▶) *T*_min_ = 0.551, *T*_max_ = 0.725
48806 measured reflections
9946 independent reflections
5931 reflections with *I* > 2σ(*I*)
*R*_int_ = 0.072

#### Refinement

*R*[*F* ^2^ > 2σ(*F* ^2^)] = 0.037
*wR*(*F* ^2^) = 0.113
*S* = 1.02
9946 reflections
490 parameters
H atoms treated by a mixture of independent and constrained refinement
Δρ_max_ = 2.07 e Å^−3^
Δρ_min_ = −0.60 e Å^−3^

### 

Data collection: *EXPOSE* in *IPDS Software* (Stoe & Cie, 1999 ▶); cell refinement: *CELL* in *IPDS Software*; data reduction: *INTEGRATE* in *IPDS Software*; program(s) used to solve structure: *SHELXS97* (Sheldrick, 2008 ▶); program(s) used to refine structure: *SHELXL97* (Sheldrick, 2008 ▶); molecular graphics: *ORTEP-3 for Windows* (Farrugia, 1997 ▶); software used to prepare material for publication: *SHELXL97*, *WinGX* (Farrugia, 1999 ▶) and *publCIF* (Westrip, 2010 ▶).

## Supplementary Material

Crystal structure: contains datablocks global, I. DOI: 10.1107/S1600536810052426/fj2374sup1.cif

Structure factors: contains datablocks I. DOI: 10.1107/S1600536810052426/fj2374Isup2.hkl

Additional supplementary materials:  crystallographic information; 3D view; checkCIF report

## Figures and Tables

**Table 1:** Hydrogen-bond geometry (Å, °)

*D*—H⋯*A*	*D*—H	H⋯*A*	*D*⋯*A*	*D*—H⋯*A*
C2—H2⋯O1^i^	0.93	2.32	3.040 (8)	134

Symmetry code: (i) 
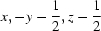
.

**Fig. 1.**
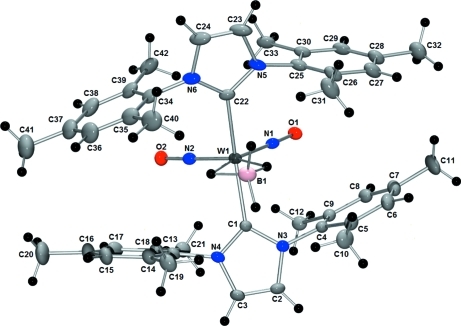
View of the title compound showing the labeling of the non-H atoms and 30% probability ellipsoids.

## supplementary crystallographic information

### Comment

In the course of our efforts on the synthesis of novel dinitrosyl hydride and dihydride tungsten derivatives bearing sterically demanding and highly donating phosphine ligands or N-heterocyclic (NHC) carbene ligands (Fraga-Hernández, 2007), the title compound, C_42_H_52_BN_6_O_2_W, (I), was synthesized as an intermediate species.

The reaction of the recently reported compound W(NO)_2_Cl_2_(IMes)_2_ (Fraga-Hernández, 2011) with [NBu_4_][BH_4_] in THF furnished the title complex W(NO)_2_(IMes)_2_(η^2^-BH_4_). The one-electron reduction of the starting material to yield the title paramagnetic 19-electron neutral complex can be explained considering that [NBu_4_][BH_4_] can act as a hydride-transfer reagent, as well as a reducing agent. In (I), the oxidation number of the W atom is formally –I. Nevertheless, a density functional theory (DFT) study combined with EPR measurements (Fraga-Hernández, 2007) indicated that the unpaired electron is delocalized on the two N atoms of the nitrosyl groups (and not on the metal center) which become equivalent and the oxidation number of the W atom is in fact 0, considering BH_4_^-^ and (NO)_2_^+^.

The W metal center is coordinated by two 1,3-bis(2,4,6-trimethylphenyl)imidazol-2-ylidene (IMes) carbene ligands, two NO groups and two H atoms of an η^2^-tetrahydroborate ligand (Fig. 1). Depending on the number of coordination sites (*n*) occupied by the BH_4_^- ^ligand, the molecular structure of the title compound consists of an approximately trigonal bipyramidal arrangement of the ligands around the W atom (*n* = 1) or might be referred to a very distorted octahedral environment around the W center with the bridging H atoms filling two coordination positions (*n* = 2). In the latter case, the *trans* carbene ligands occupy axial positions, and the coplanar NO groups and bridging H atoms (r.m.s. deviation = 0.032 Å) form the equatorial plane of a *pseudo* octahedron. The two nitrosyl ligands with a N(2)—W(1)—N(1) bond angle of 100.4 (2)° are located *trans* to the bridging borohydride moiety. The N(1)—W(1)—C(1) and N(2)—W(1)—C(1) bond angles show that the carbene ligands are bent toward the bridging borohydride group (98.5 (2) and 96.1 (2)°) and away from the NO groups. This bending [C(1)—W(1)—C(22) = 158.4 (2)°] may be due to the electronic effects caused by the strong π-acceptor groups. In comparison with the dichlorido compound W(NO)_2_Cl_2_(IMes)_2_ (Fraga-Hernández, 2011) where the five-membered rings of the carbene ligands are almost perpendicular to each other, they would be coplanar in (I) without the bending. The W—N—O bond angles [177.1 (5)° for O(1)—N(1)—W(1), and 176.0 (5)° for O(2)—N(2)—W(1)] are almost linear and indicate the coordination in form of nitrosonium groups (NO^+^). In the structure the two NO ligands are equivalent and the W—N—O bond angles are not far from linearity (average of 176.6°).

In the crystal structure, molecules are connected *via* C—H···O interactions (Table 1).

### Experimental

A mixture of [W(NO)_2_Cl_2_(IMes)_2_] (90 mg, 0.097 mmol) (Fraga-Hernández, 2011) and [NBu_4_]BH_4 _(49.7 mg, 0.195 mmol) in 10 ml ether and 5 ml THF was stirred for 21 h. After this time, the black green solution was filtered over celite and dried under vacuum. The residue was extracted with 15 ml of ether/pentane (1:2) and filtered over celite again. Removal of the solvent left a dark green solid, which was extracted with pentane (3 *x* 8 ml) and dried under vacuum, affording 62 mg of the title compound (0.071 mmol, 74%). Green crystals were obtained from a pentane solution at room temperature. IR (ATR, 22°C, cm^-1^): 1597 (NO), 1537 (NO). Elemental analysis (%) calculated for C_42_H_52_BN_6_O_2_W: C (58.14), H (6.04), N (9.68); found: C (58.40), H (5.86), N (9.78).

### Refinement

The H atoms of the tetrahydroborate group were located in difference Fourier maps. Their coordinates were freely refined, except for H1D, with *U*_iso_(H) = 1.2*U*_eq_(B). All other H positions were calculated after each cycle of refinement using a riding model with C—H = 0.93 Å and *U*_iso_(H) = 1.2*U*_eq_(C) for aromatic H atoms, and with C—H = 0.96 Å and *U*_iso_(H) = 1.5*U*_eq_(C) for methyl H atoms.

### Crystal data

**Table:**

[W(BH_4_)(C_21_H_24_N_2_)_2_(NO)_2_]	*F*(000) = 1764
*M**_r_* = 867.56	*D*_x_ = 1.392 Mg m^−^^3^
Monoclinic, *P*2_1_/*c*	Mo *K*α radiation, λ = 0.71073 Å
Hall symbol: -P 2ybc	Cell parameters from 7998 reflections
*a* = 24.7322 (13) Å	θ = 2.5–28°
*b* = 11.2183 (5) Å	µ = 2.83 mm^−^^1^
*c* = 15.0522 (8) Å	*T* = 183 K
β = 97.643 (6)°	Irregular, dark green
*V* = 4139.2 (4) Å^3^	0.3 × 0.2 × 0.18 mm
*Z* = 4	

### Data collection

**Table:**

Stoe IPDS diffractometer	9946 independent reflections
Radiation source: fine-focus sealed tube	5931 reflections with *I* > 2σ(*I*)
graphite	*R*_int_ = 0.072
φ oscillation scan	θ_max_ = 28.0°, θ_min_ = 2.5°
Absorption correction: numerical (Coppens *et al.*, 1965)	*h* = −32→32
*T*_min_ = 0.551, *T*_max_ = 0.725	*k* = 0→14
48806 measured reflections	*l* = 0→19

### Refinement

**Table:**

Refinement on *F*^2^	0 restraints
Least-squares matrix: full	H atoms treated by a mixture of independent and constrained refinement
*R*[*F*^2^ > 2σ(*F*^2^)] = 0.037	*w* = 1/[σ^2^(*F*_o_^2^) + (0.0497*P*)^2^] where *P* = (*F*_o_^2^ + 2*F*_c_^2^)/3
*wR*(*F*^2^) = 0.113	(Δ/σ)_max_ = 0.001
*S* = 1.02	Δρ_max_ = 2.07 e Å^−^^3^
9946 reflections	Δρ_min_ = −0.60 e Å^−^^3^
490 parameters	

### Special details

**Table:**

Geometry. All e.s.d.'s (except the e.s.d. in the dihedral angle between two l.s. planes) are estimated using the full covariance matrix. The cell e.s.d.'s are taken into account individually in the estimation of e.s.d.'s in distances, angles and torsion angles; correlations between e.s.d.'s in cell parameters are only used when they are defined by crystal symmetry. An approximate (isotropic) treatment of cell e.s.d.'s is used for estimating e.s.d.'s involving l.s. planes.

### Fractional atomic coordinates and isotropic or equivalent isotropic displacement parameters (Å^2^)

**Table:**

	*x*	*y*	*z*	*U*_iso_*/*U*_eq_	
W1	0.252596 (11)	−0.46192 (2)	0.731784 (16)	0.03782 (8)	
B1	0.2475 (4)	−0.5990 (9)	0.6041 (6)	0.058 (2)	
H1A	0.257 (3)	−0.567 (7)	0.539 (5)	0.07*	
H1B	0.208 (3)	−0.538 (8)	0.629 (5)	0.07*	
H1C	0.244 (3)	−0.698 (8)	0.618 (5)	0.07*	
H1D	0.2798	−0.5632	0.6564	0.07*	
N1	0.3114 (2)	−0.4285 (5)	0.8134 (3)	0.0415 (12)	
O1	0.34901 (19)	−0.4039 (4)	0.8701 (3)	0.0474 (11)	
N2	0.2018 (2)	−0.3667 (5)	0.7721 (3)	0.0433 (13)	
O2	0.1703 (2)	−0.3013 (5)	0.8032 (3)	0.0550 (13)	
N3	0.3202 (2)	−0.3248 (5)	0.5926 (3)	0.0419 (12)	
N4	0.2357 (2)	−0.2813 (5)	0.5619 (3)	0.0425 (13)	
C1	0.2711 (3)	−0.3409 (5)	0.6230 (4)	0.0370 (14)	
C2	0.3145 (3)	−0.2579 (6)	0.5145 (4)	0.0492 (17)	
H2	0.3423	−0.2353	0.4821	0.059*	
C3	0.2621 (3)	−0.2324 (7)	0.4950 (4)	0.0498 (18)	
H3	0.246	−0.1896	0.4456	0.06*	
C4	0.3720 (3)	−0.3646 (6)	0.6380 (4)	0.0447 (15)	
C5	0.3978 (3)	−0.4602 (8)	0.6032 (5)	0.0566 (17)	
C6	0.4463 (4)	−0.4984 (8)	0.6517 (7)	0.072 (2)	
H6	0.4642	−0.5635	0.6308	0.086*	
C7	0.4687 (3)	−0.4438 (8)	0.7288 (6)	0.069 (2)	
C8	0.4441 (3)	−0.3442 (8)	0.7580 (5)	0.059 (2)	
H8	0.4604	−0.3042	0.8087	0.071*	
C9	0.3949 (3)	−0.3021 (7)	0.7126 (5)	0.0484 (16)	
C10	0.3752 (4)	−0.5222 (8)	0.5180 (6)	0.075 (2)	
H10A	0.3969	−0.5029	0.4718	0.112*	
H10B	0.3383	−0.4967	0.5002	0.112*	
H10C	0.3758	−0.6068	0.5277	0.112*	
C11	0.5211 (4)	−0.4909 (10)	0.7822 (8)	0.105 (4)	
H11A	0.5151	−0.5703	0.8026	0.157*	
H11B	0.5315	−0.4401	0.8329	0.157*	
H11C	0.5496	−0.4919	0.7447	0.157*	
C12	0.3676 (3)	−0.1923 (7)	0.7442 (5)	0.0548 (18)	
H12A	0.3332	−0.2141	0.7627	0.082*	
H12B	0.3616	−0.1356	0.6962	0.082*	
H12C	0.3906	−0.1576	0.7939	0.082*	
C13	0.1794 (3)	−0.2590 (7)	0.5674 (4)	0.0476 (16)	
C14	0.1395 (3)	−0.3205 (7)	0.5122 (5)	0.0569 (19)	
C15	0.0857 (3)	−0.2897 (9)	0.5158 (6)	0.071 (2)	
H15	0.0585	−0.3312	0.4799	0.085*	
C16	0.0707 (3)	−0.2005 (9)	0.5702 (7)	0.076 (2)	
C17	0.1120 (4)	−0.1411 (8)	0.6236 (5)	0.068 (2)	
H17	0.1026	−0.0814	0.6617	0.081*	
C18	0.1665 (3)	−0.1669 (7)	0.6226 (5)	0.0541 (18)	
C19	0.1535 (3)	−0.4164 (8)	0.4485 (5)	0.066 (2)	
H19A	0.1337	−0.4879	0.4582	0.099*	
H19B	0.1919	−0.4322	0.4589	0.099*	
H19C	0.1436	−0.39	0.3878	0.099*	
C20	0.0121 (4)	−0.1635 (13)	0.5706 (9)	0.117 (4)	
H20A	−0.0106	−0.2331	0.5681	0.176*	
H20B	0.0009	−0.114	0.5194	0.176*	
H20C	0.0089	−0.1197	0.6244	0.176*	
C21	0.2104 (3)	−0.0944 (7)	0.6778 (5)	0.062 (2)	
H21A	0.23	−0.1443	0.7229	0.092*	
H21B	0.1939	−0.0296	0.7061	0.092*	
H21C	0.2351	−0.0633	0.6395	0.092*	
N5	0.2647 (3)	−0.7156 (5)	0.8370 (3)	0.0529 (15)	
N6	0.1797 (3)	−0.6770 (6)	0.7943 (4)	0.0559 (16)	
C22	0.2305 (3)	−0.6296 (6)	0.7970 (4)	0.0448 (16)	
C23	0.2356 (5)	−0.8127 (8)	0.8591 (5)	0.077 (3)	
H23	0.2498	−0.8814	0.8878	0.092*	
C24	0.1834 (4)	−0.7903 (8)	0.8319 (5)	0.074 (3)	
H24	0.1544	−0.8414	0.8371	0.089*	
C25	0.3226 (3)	−0.7031 (6)	0.8652 (4)	0.0523 (18)	
C26	0.3584 (4)	−0.7573 (7)	0.8163 (5)	0.060 (2)	
C27	0.4138 (4)	−0.7520 (8)	0.8493 (5)	0.067 (2)	
H27	0.4389	−0.7861	0.816	0.08*	
C28	0.4325 (4)	−0.6977 (8)	0.9301 (5)	0.063 (2)	
C29	0.3953 (3)	−0.6464 (7)	0.9765 (4)	0.058 (2)	
H29	0.4078	−0.6094	1.0307	0.07*	
C30	0.3397 (3)	−0.6459 (6)	0.9476 (4)	0.0510 (18)	
C31	0.3403 (4)	−0.8205 (9)	0.7283 (6)	0.081 (3)	
H31A	0.3033	−0.7989	0.7068	0.122*	
H31B	0.3426	−0.9052	0.7374	0.122*	
H31C	0.3636	−0.7976	0.6851	0.122*	
C32	0.4925 (4)	−0.7018 (10)	0.9671 (6)	0.084 (3)	
H32A	0.5136	−0.7165	0.9191	0.127*	
H32B	0.4987	−0.7646	1.0106	0.127*	
H32C	0.5032	−0.627	0.995	0.127*	
C33	0.2994 (4)	−0.5898 (7)	1.0002 (4)	0.062 (2)	
H33A	0.3184	−0.5494	1.0513	0.093*	
H33B	0.2763	−0.6503	1.0201	0.093*	
H33C	0.2776	−0.5335	0.9632	0.093*	
C34	0.1293 (3)	−0.6155 (8)	0.7676 (5)	0.0575 (19)	
C35	0.0990 (4)	−0.6413 (9)	0.6850 (6)	0.074 (3)	
C36	0.0511 (4)	−0.5790 (11)	0.6639 (7)	0.089 (3)	
H36	0.0302	−0.5941	0.609	0.106*	
C37	0.0321 (4)	−0.4949 (11)	0.7199 (8)	0.092 (3)	
C38	0.0629 (4)	−0.4782 (10)	0.8025 (7)	0.082 (3)	
H38	0.0504	−0.4247	0.8423	0.098*	
C39	0.1115 (3)	−0.5372 (10)	0.8290 (5)	0.068 (2)	
C40	0.1164 (4)	−0.7322 (10)	0.6218 (6)	0.089 (3)	
H40A	0.1178	−0.8093	0.6497	0.133*	
H40B	0.1518	−0.7118	0.6071	0.133*	
H40C	0.0907	−0.7337	0.5681	0.133*	
C41	−0.0214 (5)	−0.4306 (13)	0.6941 (10)	0.129 (5)	
H41A	−0.0506	−0.4773	0.7121	0.193*	
H41B	−0.0274	−0.4193	0.6303	0.193*	
H41C	−0.0202	−0.3545	0.7234	0.193*	
C42	0.1424 (4)	−0.5191 (10)	0.9213 (5)	0.080 (3)	
H42A	0.1396	−0.5896	0.9565	0.12*	
H42B	0.1271	−0.4526	0.9496	0.12*	
H42C	0.18	−0.5035	0.9165	0.12*	

### Atomic displacement parameters (Å^2^)

**Table:**

	*U*^11^	*U*^22^	*U*^33^	*U*^12^	*U*^13^	*U*^23^
W1	0.04481 (14)	0.03601 (12)	0.03189 (11)	−0.00153 (16)	0.00228 (8)	−0.00023 (13)
B1	0.080 (7)	0.055 (5)	0.037 (4)	−0.007 (5)	0.000 (4)	−0.012 (4)
N1	0.051 (3)	0.037 (3)	0.037 (3)	−0.004 (2)	0.010 (2)	0.003 (2)
O1	0.053 (3)	0.051 (3)	0.036 (2)	−0.005 (2)	−0.005 (2)	−0.002 (2)
N2	0.045 (3)	0.046 (3)	0.036 (3)	−0.012 (3)	−0.004 (2)	0.000 (2)
O2	0.053 (3)	0.063 (3)	0.050 (3)	0.011 (3)	0.012 (2)	−0.007 (2)
N3	0.045 (3)	0.042 (3)	0.039 (3)	0.003 (2)	0.007 (2)	0.004 (2)
N4	0.052 (3)	0.042 (3)	0.032 (3)	0.006 (3)	0.002 (2)	0.004 (2)
C1	0.042 (4)	0.035 (3)	0.034 (3)	0.004 (3)	0.006 (3)	0.001 (2)
C2	0.062 (5)	0.049 (4)	0.038 (3)	0.002 (4)	0.015 (3)	0.008 (3)
C3	0.059 (5)	0.055 (5)	0.034 (3)	0.005 (4)	0.003 (3)	0.010 (3)
C4	0.043 (4)	0.047 (4)	0.045 (3)	0.004 (3)	0.011 (3)	0.009 (3)
C5	0.053 (4)	0.049 (4)	0.070 (4)	0.001 (4)	0.017 (3)	−0.002 (4)
C6	0.055 (5)	0.058 (5)	0.105 (7)	0.012 (4)	0.020 (5)	0.011 (4)
C7	0.046 (4)	0.062 (6)	0.098 (6)	0.003 (4)	0.006 (4)	0.017 (5)
C8	0.045 (4)	0.069 (5)	0.063 (4)	−0.009 (4)	0.000 (3)	0.012 (4)
C9	0.045 (4)	0.046 (4)	0.054 (4)	−0.002 (3)	0.009 (3)	0.006 (3)
C10	0.083 (6)	0.061 (6)	0.084 (6)	0.010 (5)	0.023 (5)	−0.020 (5)
C11	0.051 (5)	0.100 (9)	0.154 (10)	0.011 (5)	−0.019 (5)	0.037 (7)
C12	0.059 (5)	0.047 (4)	0.057 (4)	−0.011 (4)	0.000 (3)	−0.005 (3)
C13	0.048 (4)	0.051 (4)	0.043 (4)	0.014 (3)	0.004 (3)	0.012 (3)
C14	0.051 (4)	0.062 (5)	0.054 (4)	0.005 (4)	−0.005 (3)	0.003 (4)
C15	0.045 (5)	0.088 (7)	0.076 (5)	0.007 (4)	−0.007 (4)	0.005 (5)
C16	0.049 (5)	0.084 (7)	0.093 (6)	0.015 (5)	0.004 (4)	0.004 (5)
C17	0.081 (6)	0.063 (5)	0.062 (5)	0.026 (5)	0.021 (4)	0.002 (4)
C18	0.060 (5)	0.050 (4)	0.053 (4)	0.013 (4)	0.010 (3)	0.005 (3)
C19	0.069 (5)	0.059 (5)	0.065 (5)	−0.002 (4)	−0.013 (4)	−0.011 (4)
C20	0.059 (6)	0.133 (12)	0.160 (11)	0.030 (7)	0.016 (6)	0.008 (9)
C21	0.073 (5)	0.046 (4)	0.067 (5)	0.009 (4)	0.014 (4)	−0.009 (4)
N5	0.075 (4)	0.038 (3)	0.042 (3)	−0.005 (3)	−0.007 (3)	0.010 (2)
N6	0.072 (4)	0.049 (4)	0.044 (3)	−0.022 (3)	−0.004 (3)	0.007 (3)
C22	0.064 (4)	0.038 (4)	0.033 (3)	−0.012 (3)	0.007 (3)	0.001 (3)
C23	0.118 (8)	0.051 (5)	0.055 (5)	−0.024 (5)	−0.009 (5)	0.016 (4)
C24	0.096 (7)	0.059 (5)	0.062 (5)	−0.039 (5)	−0.006 (5)	0.012 (4)
C25	0.067 (5)	0.039 (4)	0.046 (4)	−0.004 (4)	−0.009 (3)	0.009 (3)
C26	0.081 (6)	0.046 (4)	0.048 (4)	0.013 (4)	−0.014 (4)	−0.002 (3)
C27	0.083 (6)	0.060 (5)	0.054 (4)	0.020 (5)	−0.001 (4)	0.004 (4)
C28	0.073 (5)	0.060 (5)	0.051 (4)	0.011 (4)	−0.011 (4)	0.007 (4)
C29	0.080 (6)	0.051 (5)	0.038 (4)	−0.007 (4)	−0.013 (4)	0.006 (3)
C30	0.076 (5)	0.039 (4)	0.035 (3)	0.000 (4)	−0.002 (3)	0.007 (3)
C31	0.091 (7)	0.075 (6)	0.070 (5)	0.023 (5)	−0.017 (5)	−0.024 (5)
C32	0.077 (6)	0.091 (7)	0.078 (6)	0.011 (6)	−0.014 (5)	0.006 (5)
C33	0.089 (6)	0.060 (5)	0.034 (3)	−0.010 (4)	−0.003 (3)	0.005 (3)
C34	0.051 (4)	0.060 (5)	0.061 (4)	−0.023 (4)	0.005 (3)	0.003 (4)
C35	0.067 (6)	0.083 (7)	0.067 (5)	−0.027 (5)	−0.003 (4)	−0.003 (5)
C36	0.064 (6)	0.110 (9)	0.086 (6)	−0.021 (6)	−0.013 (5)	−0.011 (6)
C37	0.052 (5)	0.101 (9)	0.120 (9)	−0.023 (5)	0.003 (5)	0.007 (7)
C38	0.067 (6)	0.089 (7)	0.094 (6)	−0.029 (6)	0.026 (5)	−0.014 (6)
C39	0.054 (5)	0.086 (6)	0.065 (5)	−0.032 (5)	0.013 (4)	−0.002 (5)
C40	0.085 (7)	0.100 (8)	0.075 (6)	−0.028 (6)	−0.016 (5)	−0.016 (5)
C41	0.068 (7)	0.136 (14)	0.177 (12)	0.001 (7)	−0.001 (7)	0.010 (9)
C42	0.085 (6)	0.104 (8)	0.055 (4)	−0.027 (6)	0.025 (4)	−0.012 (5)

### Geometric parameters (Å, °)

**Table:**

W1—N1	1.813 (5)	C20—H20A	0.96
W1—N2	1.814 (6)	C20—H20B	0.96
W1—C1	2.221 (6)	C20—H20C	0.96
W1—C22	2.223 (6)	C21—H21A	0.96
W1—B1	2.451 (8)	C21—H21B	0.96
W1—H1B	1.98 (8)	C21—H21C	0.96
W1—H1D	1.8	N5—C22	1.371 (9)
B1—H1A	1.11 (8)	N5—C23	1.371 (10)
B1—H1B	1.29 (8)	N5—C25	1.443 (10)
B1—H1C	1.13 (9)	N6—C22	1.358 (9)
B1—H1D	1.12	N6—C24	1.389 (10)
N1—O1	1.209 (6)	N6—C34	1.436 (10)
N2—O2	1.207 (7)	C23—C24	1.324 (13)
N3—C1	1.366 (8)	C23—H23	0.93
N3—C2	1.385 (8)	C24—H24	0.93
N3—C4	1.440 (8)	C25—C26	1.368 (11)
N4—C1	1.359 (8)	C25—C30	1.411 (9)
N4—C3	1.384 (9)	C26—C27	1.395 (12)
N4—C13	1.428 (9)	C26—C31	1.516 (10)
C2—C3	1.322 (10)	C27—C28	1.383 (11)
C2—H2	0.93	C27—H27	0.93
C3—H3	0.93	C28—C29	1.356 (12)
C4—C9	1.379 (10)	C28—C32	1.515 (12)
C4—C5	1.386 (10)	C29—C30	1.387 (11)
C5—C6	1.386 (11)	C29—H29	0.93
C5—C10	1.500 (11)	C30—C33	1.492 (11)
C6—C7	1.363 (13)	C31—H31A	0.96
C6—H6	0.93	C31—H31B	0.96
C7—C8	1.372 (12)	C31—H31C	0.96
C7—C11	1.526 (11)	C32—H32A	0.96
C8—C9	1.396 (10)	C32—H32B	0.96
C8—H8	0.93	C32—H32C	0.96
C9—C12	1.512 (10)	C33—H33A	0.96
C10—H10A	0.96	C33—H33B	0.96
C10—H10B	0.96	C33—H33C	0.96
C10—H10C	0.96	C34—C39	1.387 (12)
C11—H11A	0.96	C34—C35	1.394 (11)
C11—H11B	0.96	C35—C36	1.377 (14)
C11—H11C	0.96	C35—C40	1.496 (14)
C12—H12A	0.96	C36—C37	1.387 (15)
C12—H12B	0.96	C36—H36	0.93
C12—H12C	0.96	C37—C38	1.382 (14)
C13—C14	1.386 (10)	C37—C41	1.512 (15)
C13—C18	1.389 (10)	C38—C39	1.384 (13)
C14—C15	1.380 (11)	C38—H38	0.93
C14—C19	1.512 (11)	C39—C42	1.507 (11)
C15—C16	1.375 (13)	C40—H40A	0.96
C15—H15	0.93	C40—H40B	0.96
C16—C17	1.384 (13)	C40—H40C	0.96
C16—C20	1.508 (13)	C41—H41A	0.96
C17—C18	1.382 (11)	C41—H41B	0.96
C17—H17	0.93	C41—H41C	0.96
C18—C21	1.514 (11)	C42—H42A	0.96
C19—H19A	0.96	C42—H42B	0.96
C19—H19B	0.96	C42—H42C	0.96
C19—H19C	0.96		
			
N1—W1—N2	100.4 (2)	H19A—C19—H19C	109.5
N1—W1—C1	98.5 (2)	H19B—C19—H19C	109.5
N2—W1—C1	96.1 (2)	C16—C20—H20A	109.5
N1—W1—C22	95.6 (2)	C16—C20—H20B	109.5
N2—W1—C22	97.3 (3)	H20A—C20—H20B	109.5
C1—W1—C22	158.4 (2)	C16—C20—H20C	109.5
N1—W1—B1	127.9 (3)	H20A—C20—H20C	109.5
N2—W1—B1	131.7 (3)	H20B—C20—H20C	109.5
C1—W1—B1	78.4 (3)	C18—C21—H21A	109.5
C22—W1—B1	80.0 (3)	C18—C21—H21B	109.5
N1—W1—H1B	159 (2)	H21A—C21—H21B	109.5
N2—W1—H1B	100 (2)	C18—C21—H21C	109.5
C1—W1—H1B	80 (2)	H21A—C21—H21C	109.5
C22—W1—H1B	81 (2)	H21B—C21—H21C	109.5
N1—W1—H1D	103	C22—N5—C23	110.5 (7)
N2—W1—H1D	156	C22—N5—C25	126.3 (6)
C1—W1—H1D	78	C23—N5—C25	122.6 (7)
C22—W1—H1D	83	C22—N6—C24	109.7 (7)
H1B—W1—H1D	57	C22—N6—C34	125.8 (6)
W1—B1—H1A	120 (4)	C24—N6—C34	124.1 (7)
H1A—B1—H1B	110 (5)	N6—C22—N5	104.6 (6)
W1—B1—H1C	118 (4)	N6—C22—W1	126.8 (5)
H1A—B1—H1C	121 (6)	N5—C22—W1	128.0 (5)
H1B—B1—H1C	112 (6)	C24—C23—N5	107.3 (8)
H1A—B1—H1D	107	C24—C23—H23	126.3
H1B—B1—H1D	96	N5—C23—H23	126.3
H1C—B1—H1D	107	C23—C24—N6	107.9 (7)
O1—N1—W1	177.1 (5)	C23—C24—H24	126.1
O2—N2—W1	176.0 (5)	N6—C24—H24	126.1
C1—N3—C2	111.2 (5)	C26—C25—C30	122.4 (7)
C1—N3—C4	125.0 (5)	C26—C25—N5	119.1 (6)
C2—N3—C4	123.7 (5)	C30—C25—N5	118.2 (7)
C1—N4—C3	111.3 (5)	C25—C26—C27	117.7 (7)
C1—N4—C13	126.3 (5)	C25—C26—C31	122.8 (8)
C3—N4—C13	122.1 (5)	C27—C26—C31	119.5 (8)
N4—C1—N3	103.3 (5)	C28—C27—C26	122.0 (8)
N4—C1—W1	128.5 (5)	C28—C27—H27	119
N3—C1—W1	127.4 (4)	C26—C27—H27	119
C3—C2—N3	107.0 (6)	C29—C28—C27	118.0 (8)
C3—C2—H2	126.5	C29—C28—C32	121.4 (7)
N3—C2—H2	126.5	C27—C28—C32	120.5 (8)
C2—C3—N4	107.3 (6)	C28—C29—C30	123.5 (7)
C2—C3—H3	126.4	C28—C29—H29	118.2
N4—C3—H3	126.4	C30—C29—H29	118.2
C9—C4—C5	122.8 (6)	C29—C30—C25	116.3 (7)
C9—C4—N3	118.2 (6)	C29—C30—C33	122.7 (6)
C5—C4—N3	119.0 (6)	C25—C30—C33	121.0 (7)
C6—C5—C4	116.7 (7)	C26—C31—H31A	109.5
C6—C5—C10	120.3 (8)	C26—C31—H31B	109.5
C4—C5—C10	122.9 (7)	H31A—C31—H31B	109.5
C7—C6—C5	122.3 (8)	C26—C31—H31C	109.5
C7—C6—H6	118.9	H31A—C31—H31C	109.5
C5—C6—H6	118.9	H31B—C31—H31C	109.5
C6—C7—C8	119.5 (8)	C28—C32—H32A	109.5
C6—C7—C11	120.9 (9)	C28—C32—H32B	109.5
C8—C7—C11	119.7 (9)	H32A—C32—H32B	109.5
C7—C8—C9	120.8 (8)	C28—C32—H32C	109.5
C7—C8—H8	119.6	H32A—C32—H32C	109.5
C9—C8—H8	119.6	H32B—C32—H32C	109.5
C4—C9—C8	117.6 (7)	C30—C33—H33A	109.5
C4—C9—C12	121.2 (6)	C30—C33—H33B	109.5
C8—C9—C12	121.2 (7)	H33A—C33—H33B	109.5
C5—C10—H10A	109.5	C30—C33—H33C	109.5
C5—C10—H10B	109.5	H33A—C33—H33C	109.5
H10A—C10—H10B	109.5	H33B—C33—H33C	109.5
C5—C10—H10C	109.5	C39—C34—C35	123.1 (8)
H10A—C10—H10C	109.5	C39—C34—N6	117.2 (7)
H10B—C10—H10C	109.5	C35—C34—N6	119.6 (8)
C7—C11—H11A	109.5	C36—C35—C34	116.3 (9)
C7—C11—H11B	109.5	C36—C35—C40	120.8 (8)
H11A—C11—H11B	109.5	C34—C35—C40	122.9 (9)
C7—C11—H11C	109.5	C35—C36—C37	123.7 (9)
H11A—C11—H11C	109.5	C35—C36—H36	118.1
H11B—C11—H11C	109.5	C37—C36—H36	118.1
C9—C12—H12A	109.5	C38—C37—C36	116.6 (10)
C9—C12—H12B	109.5	C38—C37—C41	121.7 (12)
H12A—C12—H12B	109.5	C36—C37—C41	121.5 (11)
C9—C12—H12C	109.5	C37—C38—C39	123.3 (10)
H12A—C12—H12C	109.5	C37—C38—H38	118.4
H12B—C12—H12C	109.5	C39—C38—H38	118.4
C14—C13—C18	121.7 (7)	C38—C39—C34	116.8 (8)
C14—C13—N4	120.0 (6)	C38—C39—C42	121.0 (9)
C18—C13—N4	117.9 (6)	C34—C39—C42	122.3 (9)
C15—C14—C13	117.7 (8)	C35—C40—H40A	109.5
C15—C14—C19	120.3 (7)	C35—C40—H40B	109.5
C13—C14—C19	121.9 (7)	H40A—C40—H40B	109.5
C16—C15—C14	122.9 (8)	C35—C40—H40C	109.5
C16—C15—H15	118.6	H40A—C40—H40C	109.5
C14—C15—H15	118.6	H40B—C40—H40C	109.5
C15—C16—C17	117.4 (8)	C37—C41—H41A	109.5
C15—C16—C20	122.6 (9)	C37—C41—H41B	109.5
C17—C16—C20	120.0 (10)	H41A—C41—H41B	109.5
C18—C17—C16	122.5 (8)	C37—C41—H41C	109.5
C18—C17—H17	118.7	H41A—C41—H41C	109.5
C16—C17—H17	118.7	H41B—C41—H41C	109.5
C17—C18—C13	117.7 (7)	C39—C42—H42A	109.5
C17—C18—C21	120.7 (7)	C39—C42—H42B	109.5
C13—C18—C21	121.5 (7)	H42A—C42—H42B	109.5
C14—C19—H19A	109.5	C39—C42—H42C	109.5
C14—C19—H19B	109.5	H42A—C42—H42C	109.5
H19A—C19—H19B	109.5	H42B—C42—H42C	109.5
C14—C19—H19C	109.5		

### Hydrogen-bond geometry (Å, °)

**Table:**

*D*—H···*A*	*D*—H	H···*A*	*D*···*A*	*D*—H···*A*
C2—H2···O1^i^	0.93	2.32	3.040 (8)	134

Symmetry codes: (i) *x*, −*y*−1/2, *z*−1/2.
